# Supplementary material for: Neurodevelopment in Normocephalic Children Exposed to Zika Virus in Utero with No Observable Defects at Birth: A Systematic Review with Meta-Analysis
Source: Int J Environ Res Public Health. 2022 Jun 14;19(12):7319. doi: 10.3390/ijerph19127319 (PMC9223424; doi:10.3390/ijerph19127319)
Supplement: Supplementary file 1 [file ijerph-19-07319-s001.zip › ijerph-1705777-SI.pdf]

Supplementary material

Table S1. Systematic review search strategy.

Search number	Query	Filters	MEDLINE number of articles
#1	Zika OR ZIKV	(""Zika""[All Fields] OR ""ZIKV""[All Fields]) AND (humans[Filter]) (1000/1/1:2021/3/1[pdat])	6,373
#2	Child OR children OR infant	(""child""[Journal] OR ""child""[All Fields] OR ""children""[Journal] OR ""children basel""[Journal] OR ""children""[All Fields] OR ""infant""[All Fields]) AND (humans[Filter]) (1000/1/1:2021/3/1[pdat])	3,001,567
#3	neurodevelopment OR neurodevelopmental OR development OR neurodevelopmental OR Bayley OR BSID	(""neurodevelopment""[All Fields] OR ""neurodevelopmental""[All Fields] OR ""development""[Journal] OR ""development rome""[Journal] OR ""development""[All Fields] OR ""neurodevelopmental""[All Fields] OR ""Bayley""[All Fields] OR ""BSID""[All Fields]) AND (humans[Filter]) (1000/1/1:2021/3/1[pdat])	2,994,826
#4	#1 AND #2 AND #3	#1 AND #2 AND #3	525

Table S2. Neurodevelopmental tools chosen to measure the long-term impact of prenatal ZIKV exposure in children.

Neurodevelopmental tool	Description	Domains evaluated	Studies where tool was applied
**The Bayley Scales of Infant and Toddler Development, Third Edition (BSID-III)**	This scale provides scores for three major developmental domains (motor, cognition, and language), varying from 40 to 160. Scores between 85 and 115 indicate normal development, while scores below 85 (-1 SD) indicate a developmental delay in the domain evaluated. Deviations in an individual's composite score fromthe normative mean (100±15) are used to classify neurodevelopment as normal: within 1 SD of the mean (≥85); mild: –1 to –2 SD classify neurodevelopment as normal: within 1 SD of the mean (≥85); mild: –1 to –2 SD (≥70 and <85); moderate: –2 to –3 SD (≥55 1 SD of the mean (≥85); mild: –1 to –2 SD (≥70 and <85); moderate: –2 to –3 SD (≥55 and <70); or severe: more than 3 SD below the standard mean scores (<55). This tool is cross-culturally validated for its use with the Brazilian population.	1. Cognitive<br>2. Receptive language<br>3. Expressive language<br>4. Gross motor<br>5. Fine motor	Lopes Moreira ME., et al; Einspieler C., et al; Nielsen-Saines K., et al; Rodrigues Gerzson L. et al; Faiçal AV., et al; Peçanha., et al; Sobhani NC., et al; Cranston JS., et al; Coutinho CM., et al; Abtibol-Bernardino MR., et al; Marbán Castro E., et al.
**The Prechtl's General Movement Assessment**	This tool has been considered a breakthrough in the prediction of neurodevelopmental impairment, mainly in the fidgety age. It is a quick, non-invasive and non-intrusive method that assess the quality of GMs. GMA provides excellent predictive power and has provided best evidence and sensitivity compared to brain imaging techniques. GMs in ZIKV-exposed infants were not described to date.	1. Motor	Soares-Marangoni DA., et al;
**The Alberta Infant Motor Scale (AIMS)**	It is an observational motor examination of the infant's most mature movements in prone, supine, sitting, and standing positions. It is one of the most widely used tools to assess infant motor performance from birth to independent walking. This valid, reliable, observational, performance-based tool can be particularly useful for monitoring potential gross motor delays in infants at risk for CNS dysfunction, such as ZIKV-exposed infants. It is validated in Spanish populations.	1. Gross motor	Soares-Marangoni DA., et al; Mulkey SB.; et al.

**The Denver Developmental Screening Test II [2] (DDST-II)**	It is a simple method of screening for evidence of slow development in infants and preschool children. It was standardized on 1,036 presumably normal children (from two weeks to six years of age) whose families reflect the occupational and ethnic characteristics of the population of Denver.	1. Personal social<br>2. Fine motor<br>3. Adaptive motor<br>4. Language<br>5. Gross motor	Oliveira Vianna RA., et al;<br>Pimentel R., et al.
**The Mullen Scales of Early Learning**	It is a test developed for the use in the USA as a comprehensive measure of early childhood development, to have adequate validity and sensitivity when adapted for the use in LMICs (Bangirana et al., 2014; Bodeau-Livinec et al., 2019; Bornman et al., 2018; Koura et al., 2013). Children in lower risk populations outside of the USA have been shown to perform in similar ways to the American normative sample on the MSEL (Bornman, Sevcik, Romski, & Pae, 2010). The MSEL has excellent correspondence validity with the BSID (Bradley-Johnson, 2001) and has been used with Spanish-speaking populations (Connery et al., 2019).	1. Visual reception<br>2. Receptive language<br>3. Expressive language<br>4. Gross motor<br>5. Fine motor	Valdes V., et al;<br>Familiar I., et al,
**The Warner Initial Developmental Evaluation of Adaptive and Functional Skills (WIDEA)**	It is a validated measure of multidomain development, including adaptive and functional skills. It is a 50-item-questionnaire that can be used on children up to 30 months of age; and is available in Spanish.	1. Self-care<br>2. Communica tion<br>3. Social cognition<br>4. Mobility	Mulkey SB., et al;
**The Survey of Wellbeing of Young Children**	It is a neurodevelopment and behaviour screening instrument validated for use in Brazil. The SWYC is particularly advantageous due to its ease of use as a first-line screening tool and its capacity to assess children with varying degrees of neurological impairment. It includes: a) milestones for screening cognitive, language, and motor development in children under 60 months of age, b) the Baby Pediatric Symptom Checklist (BPSC) for social/emotional screening of children	1. Cognitive<br>2. Language<br>3. Motor<br>4. Social-emotional	Da Silva PFS., et al.

	under 18 months; and c) the Preschool Pediatric Symptom Checklist (PPSC) for social/emotional screening of children aged 18 to 60 months.		
**The Fagan Test of Infant Intelligence**	It is a visual recognition test. Fagan suggested that measures of early novelty preferences could predict later intelligence by presuming that novelty preference tests tap into the same functions as those measured by later intelligence tests. With more developed visual attention for human faces, the longer infants will look at an unfamiliar face presented.	1. Cognitive	Familiar I., et al.
**The Ages and Stages Questionnaire (ASQ)**	It is a 30-item parent-reported screening test validated in France, used to identify toddlers at risk for developmental delay across five dimensions. Each dimension consists of six questions, with possible responses: “yes” (10 points) if the child performs the behaviour, “sometimes” (5 points), and “not yet” (0 points). A score for each dimension was calculated by adding the points from each dimension. Abnormal ASQ outcomes were described as a dimension score below validated cut-off values, set at 2SD below the mean using reference norms.	1. Personal-Social<br>2. Problem solving<br>3. Communication<br>4. Gross motor<br>5. Fine motor	Grant R., et al.
**The Modified Checklist for Autism on Toddlers (MCHAT)**	It is used to identify toddlers at risk for behaviour disorder. The test is parent-reported screening.	1. Behaviour	Grant R., et al.
**The French MacArthur Inventory Scales**	It is used to assess French language acquisition for which the parent identified from a validated list of 100 words the words that the toddler says. Total word count is calculated as the sum of the words the toddler says spontaneously. Abnormal IDFC outcomes are described as total word count below validated 10th percentile thresholds derived from a reference population.	1. French language	Grant R., et al.

BSID-III: Bayley Scales of Infant and Toddler Development, Third Edition; CNS: Central Nervous System; DDST: Denver Developmental Screening Test, II Edition; LMIC: Low- and middle-income countries; USA: United States of America; ZIKV: Zika virus. *The study was conducted in Spain, but women were migrants who had recently travelled to an area of risk for ZIKV (Colombia, The Dominican Republic etc.)

Table S3. Full data on the prevalence of neurodevelopmental delays in children with prenatal ZIKV exposure and study authors.

First author	Cognitive delay (<-1SD)	Number of children assessed for neurodevelopment	Prevalence	95% C.I.	% weight (fixed)	% weight (random)
**Any cognitive delay**						
**Lopes Moreira ME**	11	94	0.117	(0.067, 0.198)	19.67	18.14
**Einspieler C**	1	56	0.018	(0.003, 0.094)	11.76	13.34
**Nielsen-Saines K**	14	146	0.096	(0.058, 0.155)	30.49	22.40
**Rodrigues Gerzson L**	1	17	0.059	(0.010, 0.270)	3.64	5.41
**Peçanha**	4	84	0.048	(0.019, 0.116)	17.59	17.06
**Sobhani NC**	0	3	0.000	(0.000, 0.561)	0.73	1.22
**Faiçal AV**	4	29	0.138	(0.055, 0.306)	6.14	8.33
**Abtibol-Bernardino MR**	5	26	0.192	(0.085, 0.379)	5.52	7.65
**Marbán-Castro E**	1	21	0.048	(0.008, 0.227)	4.47	6.44
**Fixed Effects (Inverse Variance) Pooled Prevalence**	41	476	0.065	(0.041, 0.093)	100.00	.
**Random Effects (DerSimonian & Laird) Pooled Prevalence**	41	476	0.065	(0.034, 0.102)	.	100.00
**Any language delay**						
**Lopes Moreira ME**	25	94	0.266	(0.187, 0.363)	19.67	15.43
**Einspieler C**	6	56	0.107	(0.050, 0.215)	11.76	13.46
**Nielsen-Saines K**	51	146	0.349	(0.277, 0.430)	30.49	16.73
**Rodrigues Gerzson L**	9	17	0.529	(0.310, 0.738)	3.64	7.86
**Peçanha**	31	84	0.369	(0.274, 0.476)	17.59	15.05
**Sobhani NC**	0	3	0.000	(0.000, 0.561)	0.73	2.31
**Faiçal AV**	9	29	0.310	(0.173, 0.492)	6.14	10.41

**Abtibol-Bernardino MR**	10	26	0.385	(0.224, 0.575)	5.52	9.89
**Marbán-Castro E**	7	21	0.333	(0.172, 0.546)	4.47	8.86
**Fixed Effects (Inverse Variance) Pooled Prevalence**	148	476	0.297	(0.254, 0.342)	100.00	.
**Random Effects (DerSimonian & Laird) Pooled Prevalence**	148	476	0.297	(0.217, 0.382)	.	100.00
**Any motor delay**						
**Lopes Moreira ME**	18	94	0.191	(0.125, 0.283)	19.67	14.12
**Einspieler C**	1	56	0.018	(0.003, 0.094)	11.76	13.03
**Nielsen-Saines K**	24	146	0.164	(0.113, 0.233)	30.49	14.78
**Rodrigues Gerzson L**	4	17	0.235	(0.096, 0.473)	3.64	9.12
**Peçanha**	20	84	0.238	(0.160, 0.339)	17.59	13.92
**Sobhani NC**	0	3	0.000	(0.000, 0.561)	0.73	3.33
**Faiçal AV**	1	29	0.034	(0.006, 0.172)	6.14	11.08
**Abtibol-Bernardino MR**	9	26	0.346	(0.194, 0.538)	5.52	10.70
**Marbán-Castro E**	0	21	0.000	(0.000, 0.155)	4.47	9.92
**Fixed Effects (Inverse Variance) Pooled Prevalence**	77	476	0.132	(0.100, 0.168)	100.00	.
**Random Effects (DerSimonian & Laird) Pooled Prevalence**	77	476	0.115	(0.048, 0.201)	.	100.00
**Moderate and severe cognitive delay**						
**Lopes Moreira ME**	6	94	0.064	(0.030, 0.132)	23.86	19.84
**Einspieler C**	0	56	0.000	(0.000, 0.064)	14.27	16.20
**Nielsen-Saines K**	8	146	0.055	(0.028, 0.104)	36.99	22.52
**Rodrigues Gerzson L**	0	17	0.000	(0.000, 0.184)	4.42	8.02
**Sobhani NC**	0	3	0.000	(0.000, 0.561)	0.88	2.04
**Faiçal AV**	4	29	0.138	(0.055, 0.306)	7.45	11.42

**Abtibol-Bernardino MR**	0	26	0.000	(0.000, 0.129)	6.69	10.67
**Marbán-Castro E**	0	21	0.000	(0.000, 0.155)	5.43	9.29
**Fixed Effects (Inverse Variance) Pooled Prevalence**	18	392	0.019	(0.004, 0.041)	100.00	.
**Random Effects (DerSimonian & Laird) Pooled Prevalence**	18	392	0.014	(0.000, 0.049)	.	100.00
**Moderate and severe language delay**						
**Lopes Moreira ME**	10	94	0.106	(0.059, 0.185)	23.86	23.86
**Einspieler C**	3	56	0.054	(0.018, 0.146)	14.27	14.27
**Nielsen-Saines K**	17	146	0.116	(0.074, 0.179)	36.99	36.99
**Rodrigues Gerzson L**	2	17	0.118	(0.033, 0.343)	4.42	4.42
**Sobhani NC**	0	3	0.000	(0.000, 0.561)	0.88	0.88
**Faiçal AV**	2	29	0.069	(0.019, 0.220)	7.45	7.45
**Abtibol-Bernardino MR**	6	26	0.231	(0.110, 0.421)	6.69	6.69
**Marbán-Castro E**	1	21	0.048	(0.008, 0.227)	5.43	5.43
**Fixed Effects (Inverse Variance) Pooled Prevalence**	41	392	0.084	(0.054, 0.118)	100.00	.
**Random Effects (DerSimonian & Laird) Pooled Prevalence**	41	392	0.084	(0.054, 0.118)	.	100.00
**Moderate and severe motor delay**						
**Lopes Moreira ME**	7	94	0.074	(0.037, 0.146)	23.86	22.88
**Einspieler C**	1	56	0.018	(0.003, 0.094)	14.27	15.67
**Nielsen-Saines K**	7	146	0.048	(0.023, 0.096)	36.99	30.24
**Rodrigues Gerzson L**	0	17	0.000	(0.000, 0.184)	4.42	5.70
**Sobhani NC**	0	3	0.000	(0.000, 0.561)	0.88	1.22
**Faiçal AV**	0	29	0.000	(0.000, 0.117)	7.45	9.12
**Abtibol-Bernardino MR**	3	26	0.115	(0.040, 0.290)	6.69	8.30
**Marbán-Castro E**	0	21	0.000	(0.000, 0.155)	5.43	6.88

**Fixed Effects (Inverse Variance) Pooled Prevalence**	18	392	0.022	(0.006, 0.045)	100.00	.
**Random Effects (DerSimonian & Laird) Pooled Prevalence**	18	392	0.020	(0.003, 0.047)	.	100.00

Any cognitive delay: Test for heterogeneity: Q Heterogeneity chi-squared = 11.60 (d.f. = 8) p = 0.1701. I^2 (variation in prevalence attributable to heterogeneity) = 31.0%. Moment-based estimate of between-study variance Tau^2 = 0.0092. Differences between publications are low (I^2 = 31.0%), thus we should consider the fixed effects pooled prevalence.

Any language delay: Test for heterogeneity: Q Heterogeneity chi-squared = 22.77 (d.f. = 8) p = 0.0037. I^2 (variation in prevalence attributable to heterogeneity) = 64.9%. Moment-based estimate of between-study variance Tau^2 = 0.0378. Differences between publications are large (I^2 = 64.9%), thus we should consider the random effects pooled prevalence.

Any motor delay: Test for heterogeneity: Q Heterogeneity chi-squared = 37.02 (d.f. = 8) p <0.0001. I^2 (variation in prevalence attributable to heterogeneity) = 78.4%. Moment-based estimate of between-study variance Tau^2 = 0.0742. Differences between publications are large (I^2 = 78.4%), thus we should consider the random effects pooled prevalence.

Moderate and severe cognitive delay: Test for heterogeneity: Q Heterogeneity chi-squared = 13.42 (d.f. = 7) p = 0.0626. I^2 (variation in prevalence attributable to heterogeneity) = 47.8%. Moment-based estimate of between-study variance Tau^2 = 0.0210. Differences between publications are low (I^2 = 47.8%), thus we should consider the fixed effects pooled prevalence.

Moderate and severe language delay: Test for heterogeneity: Q Heterogeneity chi-squared = 6.08 (d.f. = 7) p = 0.5306. I^2 (variation in prevalence attributable to heterogeneity) = 0.0%. Moment-based estimate of between-study variance Tau^2 = 0.0000. Differences between publications are low (I^2 = 0.0%), thus we should consider the fixed effects pooled prevalence.

Moderate and severe motor delay: Test for heterogeneity: Q Heterogeneity chi-squared = 8.49 (d.f. = 7) p = 0.2918. I^2 (variation in prevalence attributable to heterogeneity) = 17.5%. Moment-based estimate of between-study variance Tau^2 = 0.0049. Differences between publications are low (I^2 = 17.5%), thus we should consider the fixed effects pooled prevalence.

Table S4. Assessment of the quality of the articles included in the meta-analysis (studies evaluating neurodevelopment with the BSID-III scale)

First author	Confounding	Selection bias	Deviation for intended intervention	Measurement of outcome	Outcome definition	Other	Certainty	Reasons for certainty assessment
Nielsen-Saines K.*							Low	CN; PS; GA; SDN; CIN; SPW.
Lopes-Moreira M.E. *							Low	CN; PS; GA; SDN; CIN; SPW.
Peçanha M.							Very Low	CN; PS; GA; SDY; RCI; SPW; BSIDN.
Einspieler C.							Moderate	SAC; PS; GA; SDN; CIN; SPW; BSIDN.
Faiçal A.V.							Very low	CN; PS; GAN; SDY; CIN; BSIDN; SPW; sample size is small (n=29).
Abtibol-Bernardino M.R.							Very low	CN; CSS; GA; SDN; CIN; SPW; sample size is small (n=26).
Marbán-Castro E.							Very low	CN; PS; GA; SDY; CIN; APW; The sample size is small (n=21).
Rodrigues Gerzson L.							Moderate	SAC; CrosSS; GA; SDY; CIY; SPW; sample size is small (n=17 non-microcephalic children exposed to ZIKV, and 20 non-microcephalic children without exposure).
Sobhani N.C.							Very low	CN; PS; GA; SDN; CIY; SPW; sample size is very small (n=3), four twin pregnancies (n=8), but only 3 infants had normal neonatal outcomes.

	Low risk
	Uncertain risk
	High risk

*In these two studies some participants might overlap. Risk of bias for "Confounding" was based on lack of data on gestational age at maternal ZIKV exposure, reporting of other congenital infections, and sociodemographic characteristics. "Selection bias" was evaluated based on the type of study (prospective vs. retrospective), and inclusion of pregnant women (symptomatic vs. all pregnant women). "Deviation for intended intervention" does not apply as studies were observational in nature. "Measurement of outcome" was evaluated based on the inclusion of a control group, and the reporting of disaggregated data for the BSID-III scale. "Outcome definition" was the same in all studies, as was defined by the tool (BSID-III). "Other" factors to evaluate risk of bias included specific items of each study, including sample size.

Legend:
APW: The study includes pregnant women, symptomatic and asymptomatic cases, and information on confirmed and probable ZIKV cases (not only laboratory confirmed cases by PCR).
BSIDN: The study does not provide disaggregated data for the range of delays resulted from the BSID-III scale.
CIN: This study does not provide information on congenital infections other than ZIKV screening.
CIY: This study provides information on congenital infections other than ZIKV screening.
CN: This study does not have a control group.
CrosSS: This is a cross-sectional study.
CSS: This is a case-series.
GA: This study accounts for gestational age at infection.
GAN: This study does not provide data on gestational age at infection.
PS: This is a prospective study
RCI: This study rules out other congenital infections.
SAC: This study includes sex- and age-matched neurotypical controls without maternal ZIKV exposure.
SDF: This study accounts on sociodemographic factors.
SDN: The study does not provide information on the sociodemographic characteristics of the population.
SDY: This study provides information on sociodemographic characteristics of the population.
SPW: The study only includes symptomatic pregnant women who seek healthcare.
